# Trace BaTiO_3_ Doping-Derived PVDF-Based Composite Thick Film for Dielectric Energy Storage

**DOI:** 10.3390/ma19061137

**Published:** 2026-03-14

**Authors:** Lixian Wang, Yangfan Zhang, Shengqi Li, Zhonghua Yao, Hua Hao, Minghe Cao, Wen Zhang, Zhijian Wang, Hanxing Liu

**Affiliations:** 1 State Key Laboratory of Silicate Materials for Architectures, School of Materials Science and Engineering, Wuhan University of Technology, Wuhan 430070, China; 345266@whut.edu.cn (L.W.); 294804@whut.edu.cn (Y.Z.); 349881@whut.edu.cn (S.L.); haohua@whut.edu.cn (H.H.); caominghe@whut.edu.cn (M.C.); lhxhp@whut.edu.cn (H.L.); 2 Sanya Science and Education Innovation Park of Wuhan University of Technology, Sanya 572000, China; 3 Wuhan Sunmoon Battery Co., Ltd., No. 231 Xingsan Road, Wuhan 430090, China; zhangwenwhut@126.com; 4 College of Mechanical Engineering, Jiaxing University, Jiaxing 314001, China; wangzj403@163.com

**Keywords:** polymer-ceramics composite, BaTiO_3_ nanoparticles, PVDF, nanocomposite

## Abstract

Ceramic-polymer nanocomposites combine the respective advantages of ceramics and polymers, boasting superior mechanical flexibility, thermal stability, optical transparency, and electrical conductivity, enabling their wide use in cutting-edge fields like medicine, aerospace, optoelectronic devices, and energy storage components. Notably, ceramic-polymer nanocomposites are a promising, widely recognized strategy for developing high-energy-density, low-dielectric-loss, and flexible capacitors, due to the ceramic phase’s intrinsic high dielectric constant, which enhances dielectric capability, and the polymer phase’s high breakdown strength and mechanical flexibility. Ultimately, ceramic-polymer nanocomposites can reach an optimal dielectric performance. In this study, polyvinylidene fluoride (PVDF) was used as the matrix material and barium titanate (BaTiO_3_) as the reinforcing phase within the composite structure. The BaTiO_3_ ceramic particles were incorporated into PVDF via spin-coating technology, with composite formulations prepared at different concentrations (0.5 wt%, 1.0 wt%, 1.5 wt%, 2.0 wt%, 2.5 wt%, 3.0 wt%). A series of key parameters were measured and compared, such as the dielectric constant, breakdown strength, and energy storage density, of the BT/PVDF nanocomposite. The results indicated that the BT/PVDF nanocomposite with the optimal low BaTiO_3_ content demonstrates remarkable performance, achieving a breakdown strength (E_b_) of 500 MV/m and an effective energy storage density of 15.5 J/cm^3^. This represents an improvement over conventional uniformly high-filler films.

## 1. Introduction

In recent years, nanocomposites with components such as polymeric and ceramic NPs have been rapidly developing for a wide range of applications due to their superior physical and chemical characteristics, including flexible electronics, flexible capacitors, embedded energy storage devices, radar systems, aerospace, and hybrid and electric vehicles [1,2,3,4]. So, a good dielectric material should have a high dielectric constant (ε_r_), an ultra-low loss tangent (tanδ), a high electrical breakdown strength (E_b_), and finally achieve high discharge energy density (U_dis_) and discharge efficiency (η) [5,6,7,8]. ceramics such as BaTiO_3_ [9,10,11], BaSrTiO_3_ [12,13,14], CaCuTi_4_O_12_ (CCTO) [15,16,17], Pb (Zr,Ti)O_3_ (PZT) [18,19,20,21], and others have high ε_r_ and low tanδ [22,23,24,25,26]. But their E_b_ is limited to ~400 kV/cm, which becomes a significant bottleneck for energy storage devices operating at high drive voltages. Polyvinylidene fluoride (PVDF) is a polymer with unique ferroelectric crystalline structures, excellent mechanical flexibility, and superior dielectric breakdown strength [27,28,29,30,31]. It attracted considerable attention in materials science research.

Obviously, ceramic materials alone cannot meet the requirement for high Eb in energy storage devices. Also, materials with high E_b,_ such as polymers, cannot meet the demand of high ε_r_ for energy storage devices. So, this study centers on a materials strategy of dispersing high-ε_r_ ceramic fillers within a flexible polymer matrix to form a composite film [2,3,4].

Researchers can lower the Curie point to room temperature by tuning the Ba/Sr ratio [12,13]. This can obtain a high ε_r_ and low tanδ. However, research shows that introducing strontium would increase material costs, and its complex chemical composition makes it difficult to achieve uniform filler diffusion within the substrate.

CCTO NPs have a ε_r_ (reaching ~10^5^, at 25 °C) and remain a broad range of temperatures and frequencies [15]. However, the mechanism responsible for these giant dielectric properties leads to high dielectric losses and leakage currents. CCTO nanocomposite materials are unsuitable under high electric fields. PZT NPs, a piezoelectric filler, are primarily used in composite materials for piezoelectric sensing and transducer applications [20,21]. However, lead is toxic to the environment, reduces the composite’s flexibility, and lowers its mechanical properties.

BT NPs have a high ε_r_ (~10^3^ at 25 °C) and a low tanδ (~10^−1^ at 25 °C). As a ferroelectric perovskite material, the BT-polymer nanocomposite is already the main part of extensive research, driven by its significant potential in the ceramic and electronic industries. This enables the composite material to remain high E_b_ while retaining the inherent flexibility of the polymer substrate. Moreover, the lead-free nature of barium titanate aligns with the electronics manufacturing industry’s growing emphasis on environmental sustainability. The product’s economic viability for large-scale commercial applications is further enhanced by established industrial production processes and relatively low raw material costs.

BT/PVDF composite materials offer significant advantages in dielectric properties, mechanical flexibility, environmental friendliness, low cost, and technological maturity, making them the most practically valuable research area in the field of flexible dielectric materials [9,10,11]. Subsequent studies will focus on furthering their performance potential through nanostructure design, precise interface control, and innovative preparation techniques, thereby advancing their practical application in next-generation flexible electronic devices.

In this work, polyvinylidene fluoride (PVDF) is selected as the polymer substrate and barium titanate (BT) as the dielectric filler material. While traditional BT/PVDF composites typically rely on high ceramic loadings (>30 wt%) to achieve high dielectric constants, this strategy often compromises the mechanical flexibility, processability, and breakdown strength (E_b_) of the polymer matrix [2,10,11]. In this work, we demonstrate a design that incorporates only a trace amount (0.5–3.0 wt%) of BT NPs into the PVDF matrix via a simple solution-casting method. At extremely low filler loading at 1.0 wt% BT, the composite exhibits optimal dielectric performance. This study aims to investigate percolation behavior and interfacial effects at dilute concentrations, rather than maximizing permittivity. As a result, the fabricated composite thick film achieves a balance between high breakdown strength and discharged energy density. The BT/PVDF composite thick film with 1.0 wt% BT NPs shows exceptional performance, with an E_b_ of 500 MV/m, U_dis_ of 15.5 J/cm^3^, and η of 68%.

## 2. Materials and Methods

### 2.1. Materials

Polyvinylidene fluoride (PVDF) was supplied by Dongguan Hao Sheng New Material Co. (Dongguan, China). N,N-dimethylformamide (DMF) of analytical reagent (AR) grade, and BaTiO_3_ NPs (particle size of 200 nm) were procured from Sinopharm Chemical Reagent Co., Ltd. (Shanghai, China) and Sinocera Co., Ltd. (Dongying, China).

### 2.2. Preparation of BaTiO_3_(BT)/PVDF Composite Films

**Figure 1:**
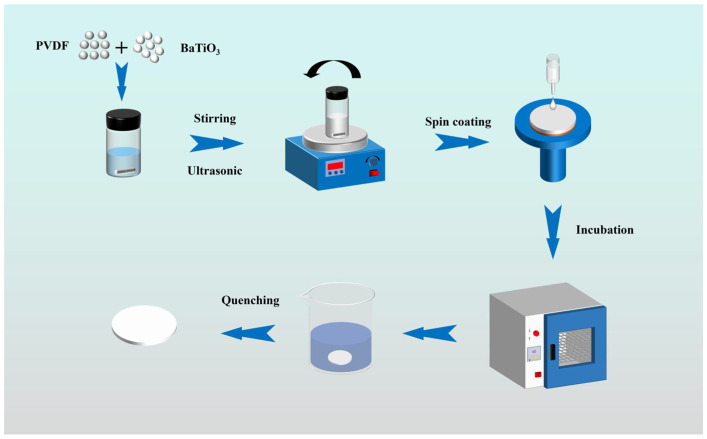
The process of preparing composite film by spin coating method.

Figure 1 illustrates the detailed experimental procedure. In this study, a spin-coating method was used to prepare BT/PVDF composite thick films (about 20 μm) through a series of processes. BT NPs with varying contents (0.5 wt%, 1.0 wt%, 1.5 wt%, 2.0 wt%, 2.5 wt%, and 3.0 wt%) were put into the DMF solution (~7 g) in a 20 mL glass container. To ensure uniform dispersion of the nanoparticles, a stepwise processing method was employed in this study. BT NPs were initially dispersed at room temperature and stirred magnetically at 1000 rpm for 30 min. Subsequently, the system received 15 min of ultrasonic treatment (power 300 W, frequency 20 kHz) to break up nanoparticle agglomerates. Stirring continued for a further 30 min to maintain system stability. Finally, a second 15 min ultrasonication was followed by 30 min of stirring. This final step proved crucial for achieving uniform dispersion of barium titanate nanoparticles within the DMF solution.

Subsequently, 1.5 g of PVDF powder was added to the solution and stirred in a water bath at 45 °C for 2 h to ensure complete dissolution of PVDF. Then, the solution was stirred at room temperature for 12 h (~1000 r/min) to promote full molecular chain stretching and enhance solution homogeneity. Then, it was ultrasonicated for 15 min to achieve a stable white composite solution with no sedimentation or delamination. The homogeneous composite solution was spin-coated onto a quartz slide and then dried at 60 °C for 6 h to ensure complete evaporation of DMF. The film was subsequently heated at 205 °C for 10 min, then immediately quenched in deionized water at 0 °C to prepare a thick-film sample of uniform thickness.

### 2.3. Characterization

Scanning electron microscopy (SEM) (7610F Plus; JEOL, Tokyo, Japan) was performed characterize the morphology. X-ray diffraction (XRD) (D8 Advance, Bruker, Karlsruhe, Germany) analysis was employed to characterize the crystalline phase composition of BT/PVDF. The characterization of the BT/PVDF composite was performed using a micro-Fourier transform infrared (FT-IR) spectroscopy system (iN10-iS50, Thermo Fisher Scientific, Waltham, MA, USA), yielding an infrared absorption spectrum within the spectral range of 1600 to 400 cm^−1^. Raman spectrograms were obtained using a laser confocal micro-Raman spectrometer (LabRAM Odyssey; Horiba Scientific, Kyoto, Japan) with 532 nm laser excitation. To measure dielectric properties, silver paste circular electrodes (2 mm in diameter, ~80 nm thick) were coated on both sides of the composite surface. The dielectric properties were measured from 100 Hz to 1 MHz using an Impedance Analyzer (E4980A; Keysight Technologies, Waltham, MA, USA), while the polarization-electric field (P-E) hysteresis loops were characterized at 100 Hz using a ferroelectric analyzer (TF Analyzer 2000E, aixACCT Systems GmbH, Aachen, Germany).

## 3. Results and Discussion

### 3.1. Microstructure

**Figure 2:**
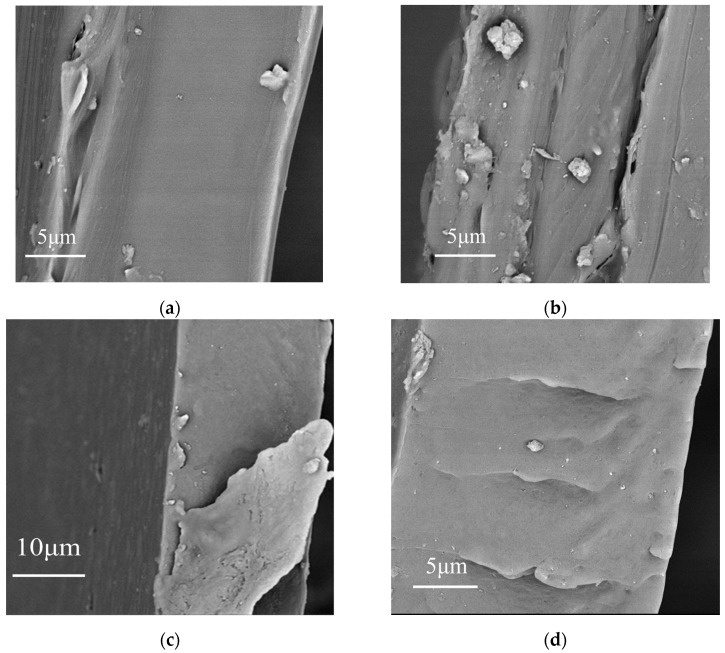

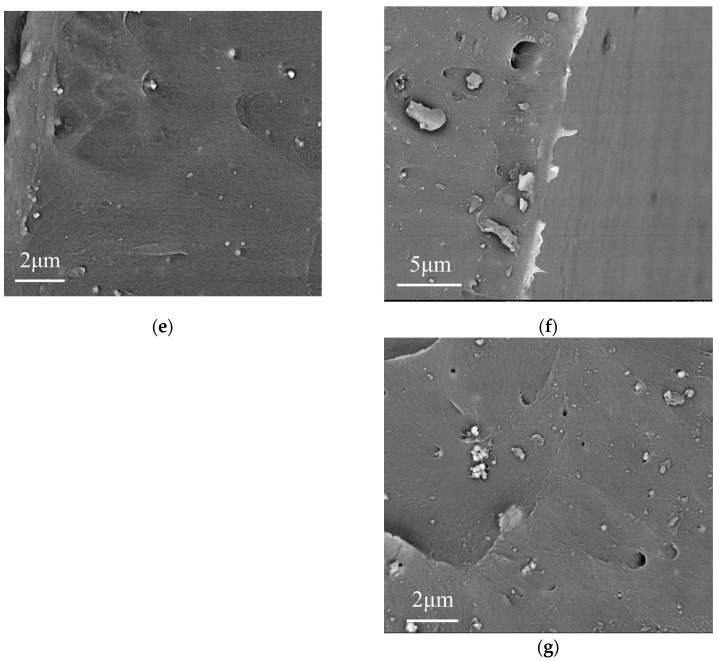
SEM images of BT/PVDF composite with different BT NPs content ((**a**–**g**): 0–3.0 wt%).

Figure 2 shows the SEM images of composites with different BT NPs content. Figure 2a–c shows that BT NPs are relatively uniformly dispersed in the PVDF without obvious defects due to the excellent dispersion and good interfacial compatibility between BT and PVDF. However, at the high loadings of 2.5 wt% and 3 wt%, as shown in Figure 2f,g, defects originating from the high loading may lead to a decline in E_b_.

### 3.2. Phase Characterization

**Figure 3:**
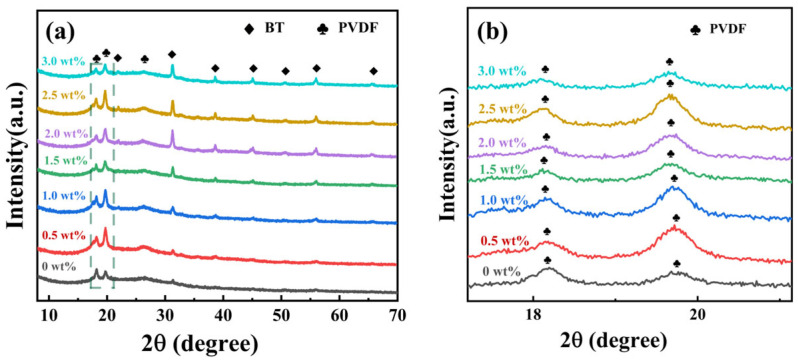
(**a**) XRD of BT/PVDF nanocomposite thick films in the 10–60° range; (**b**) XRD partial enlargement of BT/PVDF nanocomposite thick films within the area outlined by the dashed box in Figure (**a**).

Figure 3a,b show the X-ray diffraction (XRD) patterns of thick films formed by spin-coating a composite material consisting of barium titanate (BT) nanoparticles (200 nm) with a tetragonal crystal phase and polyvinylidene fluoride (PVDF). The plum blossom symbols indicate characteristic PVDF peaks, while the square symbols denote specific BT peaks. In the case of a 0 wt% BT (pure PVDF) sample, characteristic PVDF peaks are observed at 18.6°, 19.7°, and 26.5°. The 19.7° diffraction peak is a feature of (110) planes of the polar β-phase of PVDF, while the 18.6° and 26.5° peaks are attributed to the characteristic (020) and (021) planes of the non-polar α-phase. These results are consistent with previously reported findings in the literature [32]. The peak at 26.5° is relatively weak, indicating lower crystallinity of the PVDF matrix. In Figure 3b, the feature peaks of PVDF sharpen with increasing content of BT NPs at low concentrations (0.5–2.0 wt%), indicating a significant concentration dependence of the crystallization behavior in the composite membranes upon incorporation of BT NPs as reinforcing phases. This effect can be attributed to the nucleation-promoting role of BT nanoparticles at low concentrations, as previously documented in the literature [32]. This nucleation-promoting role can be explained by incorporating BT NPs as reinforcing phases into polyvinylidene fluoride (PVDF), shifting the nucleation pathway from homogeneous nucleation in pure PVDF to BT-induced heterogeneous nucleation. At low concentrations (0.5–2.0 wt%), uniformly dispersed BT NPs enhance the nucleation density and area of PVDF, thereby promoting the formation of a fine, uniform, and well-developed microcrystalline structure in the composite samples. It is proposed that the crystallinity of PVDF is influenced by the presence of BT NPs, with a degree of variation depending on the filler content. It has been shown that increasing the barium titanate content enhances the intensity of both the barium titanate and PVDF characteristic peaks. However, Figure 3b shows that the characteristic peak of PVDF is weaker compared to other concentrations at BT/PVDF with 3.0 wt% BT NPs, at 18.6° and 19.7° diffraction peaks. Excessive filler loading leads to defect formation, thereby reducing crystallinity [10]. When fillers exceed (>2.0 wt%), agglomeration of BT nanoparticles occurs, restricting crystal growth and introducing macroscopic defects such as interfacial voids, as shown in the SEM image in Figure 2, alongside microscopic defects such as lattice distortion. This results in decreased crystallinity of the composite samples and a re-broadening of XRD peak shapes. Excessive filler concentrations impede polymer chain alignment and restrict grain growth. XRD shows that the presence of BT NPs affects the crystalline structure of PVDF, with the BT/PVDF composite achieving optimal crystallinity at 1.0 wt% BT NPs.

**Figure 4:**
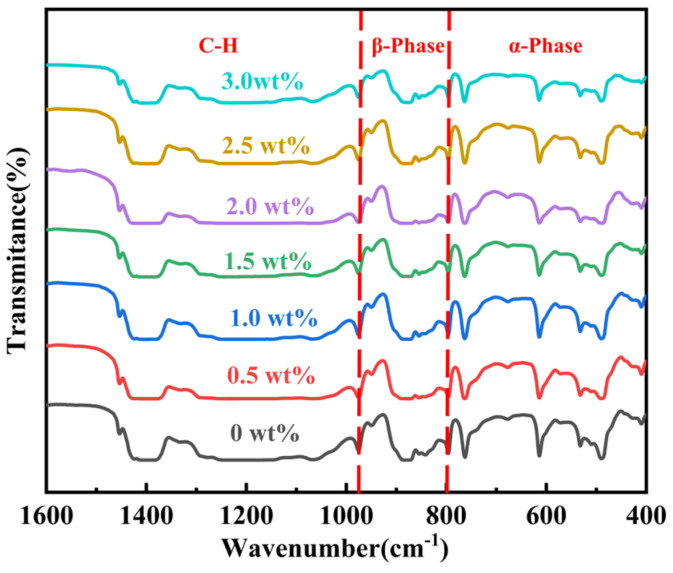
FT-IR of BT/PVDF nanocomposite thick films with varying mass.

Consequently, the X-ray diffraction pattern reveals that the BT/PVDF nanocomposite thick film exhibits only the non-polar α phase and polar β phase crystalline structure between 18° and 25°. However, the presence of other phases remains to be investigated, necessitating further analysis alongside FT-IR data. As illustrated in Figure 4, the FT-IR of BT/PVDF composite films with varying BT NPs content is presented in Table 1 for further elucidation. Table 1 shows that the composite thick films exhibit peaks at 763 cm^−1^ (the strongest), 796 cm^−1^, 976 cm^−1^, and 490, 532, 573, and 614 cm^−1^. These peaks form a complete α-phase fingerprint, indicating that the α-phase accounts for the highest proportion in the BT/PVDF nanocomposite thick films and is the main component of the samples. The absorption peaks at 610 and 768 cm^−1^ are characteristic of the non-polar α phase, whereas the peak at 844 cm^−1^ indicates a mixed phase containing β phase and possibly γ phase. However, since no γ phase conformation was detected in the combined Raman spectrum, the peaks at 510 and 844 cm^−1^ are indicative of the presence of β phase. The range of 1000–1600 cm^−1^ corresponds to the stretching vibration peaks of C-H bonds.

**Figure 5:**
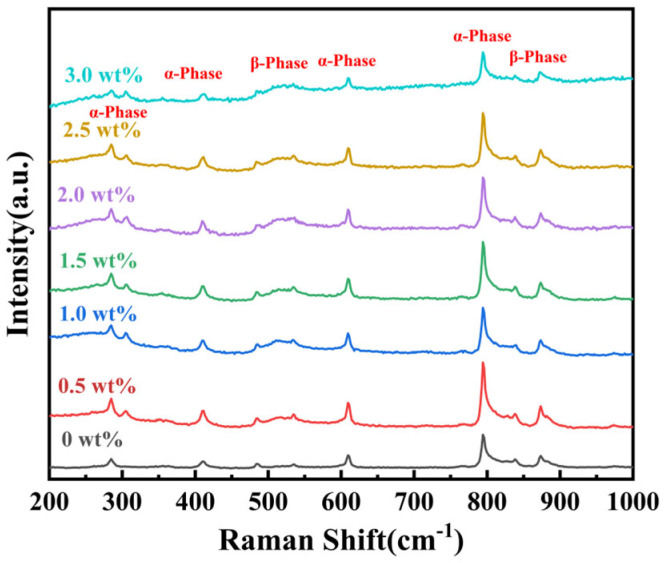
Raman spectra of BT/PVDF composite thick films with various BT NPs.

Figure 5 presents the Raman spectrum of BT/PVDF thick films with various BT NPs. By correlating Raman spectroscopic data with the conformational phase of polymer chains, the crystalline phase composition of BT/PVDF thick films is systematically elucidated. The spectral analysis reveals that characteristic Raman peak wavenumbers are distinctly associated with specific phase conformations. The peaks at 836 cm^−1^ and 536 cm^−1^ correspond to the CH_2_ rocking vibration of the β phase, indicating its conformation. This is the most characteristic peak of the β phase in the Raman spectrum, equivalent to the 840 cm^−1^ peaks observed in FT-IR. The following peaks are observed at 796 cm^−1^, 610 cm^−1^, 483 cm^−1^, 411 cm^−1^, 305 cm^−1^ and 287 cm^−1^, and these correspond to the α-phase conformation. The analysis of XRD, FT-IR, and Raman data indicates that BT/PVDF nanocomposite films predominantly exhibit α-phase and β-phase.

**Figure 6:**
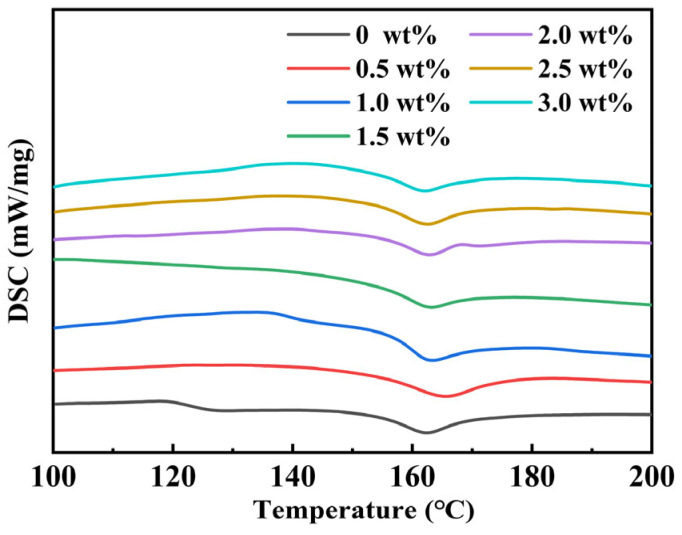
DSC curves of pure PVDF and BT/PVDF nanocomposites.

As shown in Figure 6 below, the melting peak of the PVDF matrix appears at 162 °C. The BT tetragonal-to-cubic ferroelectric phase transition peak, initially expected around 120 °C, does not appear clearly on the DSC curve. This precisely confirms the unique features of the BT core–shell structure: On the one hand, the large surface strain layer of BT, combined with its gradient structure shifting from the suppressed tetragonal phase to the cubic phase, leads to highly dispersed phase transition behavior. As a result, the faint heat flow signal is hidden within the matrix. On the other hand, the mechanical constraints caused by the PVDF matrix through the interface effectively prevent lattice changes in the BT core. This bidirectional interfacial effect has been shown to stop the latent heat of the BT phase transition from being effectively detected by DSC. In conclusion, the absence of BT phase transition peaks in DSC patterns, along with the lowered melting point of PVDF, confirms the core–shell structure of BaTiO_3_ with a strained surface layer.

### 3.3. Dielectric Properties

**Figure 7:**
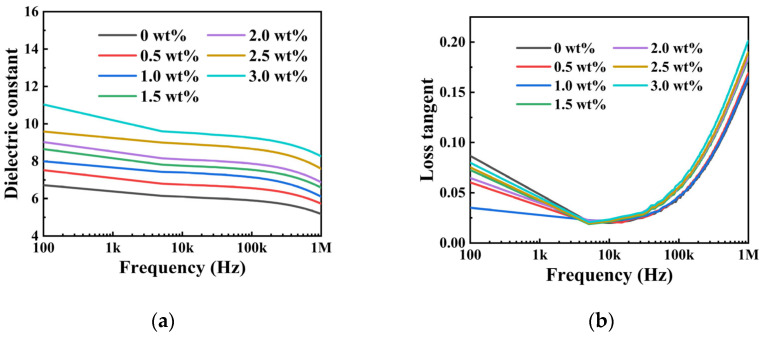

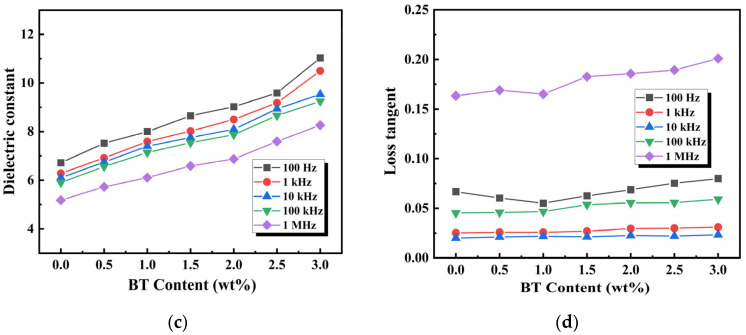
Variation of (**a**) dielectric constant and (**b**) loss with frequency for BT/PVDF nanocomposite thick films at different filler mass fractions; (**c**) dielectric constant and (**d**) loss at a series of frequencies.

Figure 7a–d show the dielectric constant (ε_r_) and loss tangent (tanδ) of a series of frequencies for BT/PVDF thick films across varying BT NPs content. Figure 7a shows that ε_r_ declines with increasing frequency. This behavior is caused by the polar ferroelectric nature of polyvinylidene fluoride, which is affected by electric dipoles. As frequency increases, the capability of dipoles, along with the electric field (E), decreases. This leads to a decrease in the dielectric constant (ε_r_) with frequency, gradually at low frequencies and more rapidly at high frequencies, where dipoles cannot keep pace with field oscillations. Figure 7b shows that the tanδ of all samples increases slightly at low frequencies but increases rapidly at high frequencies. Since the mass fraction of the filler remains constant, smaller filler dimensions result in higher filler loading, reducing interparticle spacing within the PVDF matrix. Excessive fillers increase interfacial polarization and the overall εr of the thick film, leading to agglomeration and increased dielectric loss. Figure 7c shows a gradual rise in the ε_r_ of the BT/PVDF composites as a function of the BT NPs content. For example, the dielectric constant increases from 6.7 (pure PVDF) to 11.1 (3 wt% BT/PVDF). This can be explained by the incorporation of high-ε_r_ fillers and the resulting large-polarization interfaces. These interfaces arise from the pronounced contrast in εr and electrical conductivity between the fillers and the matrix [11,33,34,35]. Figure 7d shows the frequency-dependent tanδ characteristics of PVDF-based composites with varying barium titanate (BT) weight fractions. The tanδ of all samples remains low, around 0.03 at 1 kHz. The results demonstrate that both pristine polyvinylidene fluoride (PVDF) films and BT/PVDF composite thick films exhibit significant polarization loss across the measured frequency spectrum. Obviously, the BT/PVDF composite with 1 wt% BT NPs exhibits optimal performance in dielectric tests, particularly in losses across a range of frequencies.

### 3.4. Energy Storage Performance

**Figure 8:**
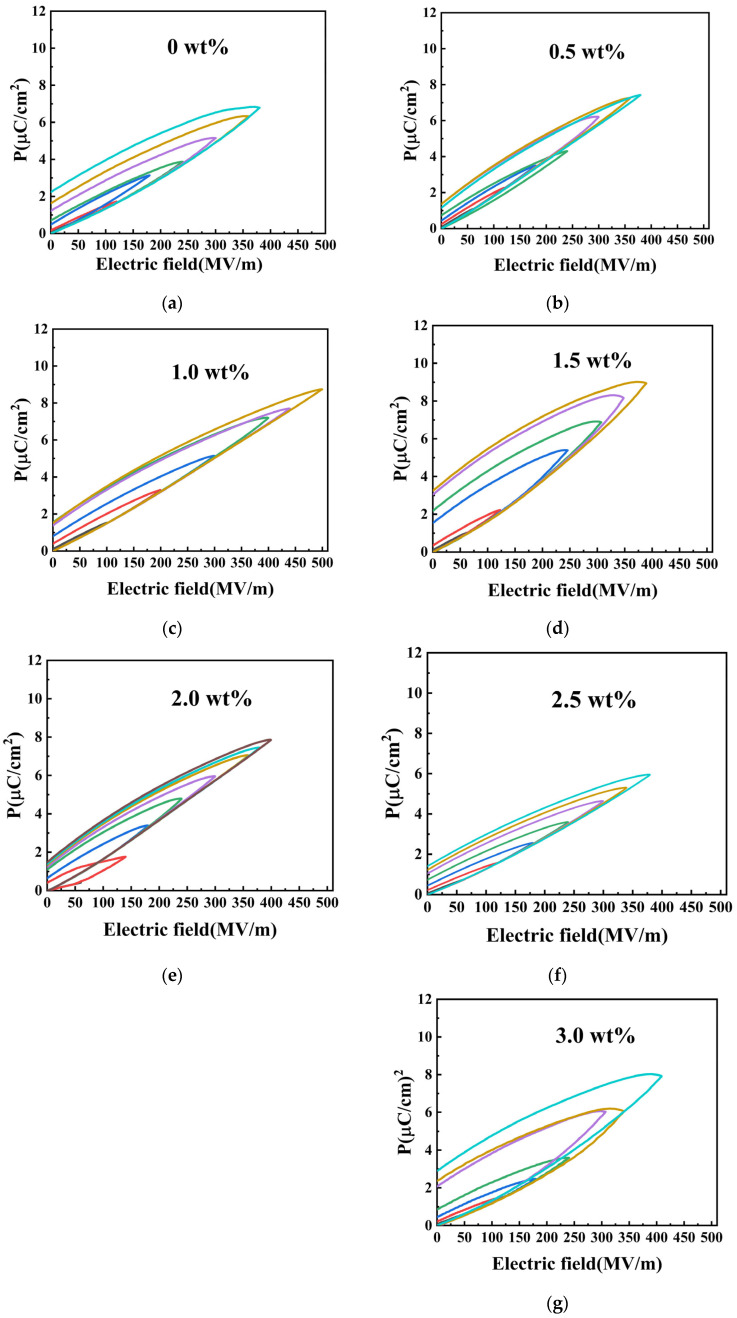
Unidirectional electric hysteresis loops of BT/PVDF thick film with varying filler mass fractions: (**a**) pure PVDF; (**b**–**g**) 0.5 wt% to 3.0 wt%.

To investigate the energy storage performance of BT/PVDF composite thick films, unipolar P-E hysteresis loops were measured for pure PVDF and composites with varying BT mass fractions at 10 Hz, as shown in Figure 8a–g. The results reveal a non-monotonic influence of BT content on energy storage performance. BT fillers introduce interfacial defects in the composite that facilitate charge transfer along the electric field direction, thereby enhancing polarization. As revealed in Figure 7, the maximum polarization (P_max_) increases with BT content. However, as the BT NPs’ mass fraction increases, excessive interfacial defects adversely affect polymer crystallinity, as reported by Merrad et al. [11], and lead to non-uniform electric field distributions at the interface. This results in an increased leakage current and a subsequent reduction in E_b_. Consequently, while the 3.0 wt% BT composite achieves high polarization, its maximum operating electric field is limited to 400 MV/m.

Energy storage performance is governed by polarization and breakdown strength, which is quantified by the difference (P_max_ − P_r_). The recoverable energy storage density (U) was calculated from the unipolar P-E loops using formula 1. The composite with 1.0 wt% BT exhibits optimal performance, sustaining a higher breakdown field (500 MV/m) and achieving a P_max_ − P_r_ of 7.2 μC/cm^2^. In contrast, the 3.0 wt% BT composite, despite its higher maximum polarization, operates at a lower field (400 MV/m) and exhibits a low P_max_ − P_r_ of 5 μC/cm^2^. This indicates that the superior polarization capability of the 1.0 wt% composite ultimately results in higher discharged energy density.

(1) U=∫Edp

In summary, energy storage capabilities are governed by the competing effects of enhanced polarization and reduced breakdown strength as BT NPs content increases, leading to an optimal filler concentration for maximum efficiency.

For practical applications, it needs a high U_dis_ and high efficiency (η). To evaluate the energy storage capabilities of the composites, a comparison of different ceramic/PVDF composites is summarized in Table 2. Table 2 shows that the dielectric performance of BT/PVDF nanocomposite thick films is significantly better than that reported in other studies. For instance, Ait Laasri et al. [34] reported an energy storage density of 9 J/cm^3^ for BT/PVDF nanocomposites, and Bi, K. et al. [35] achieved a higher value of 11.5 J/cm^3^ for similar films. In contrast, this work achieves a higher energy storage density (up to 15.5 J/cm^3^ at 500 MV/m) and energy storage efficiency (up to 68%), whilst also employing a commonly used filler type with relatively large particle size (namely 200 nm), thus demonstrating potential for practical applications.

**Figure 9:**
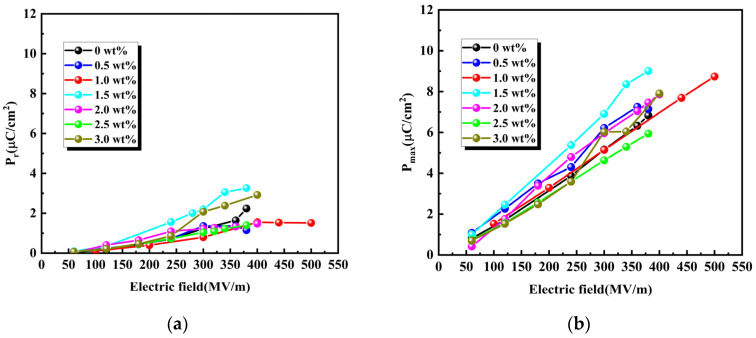

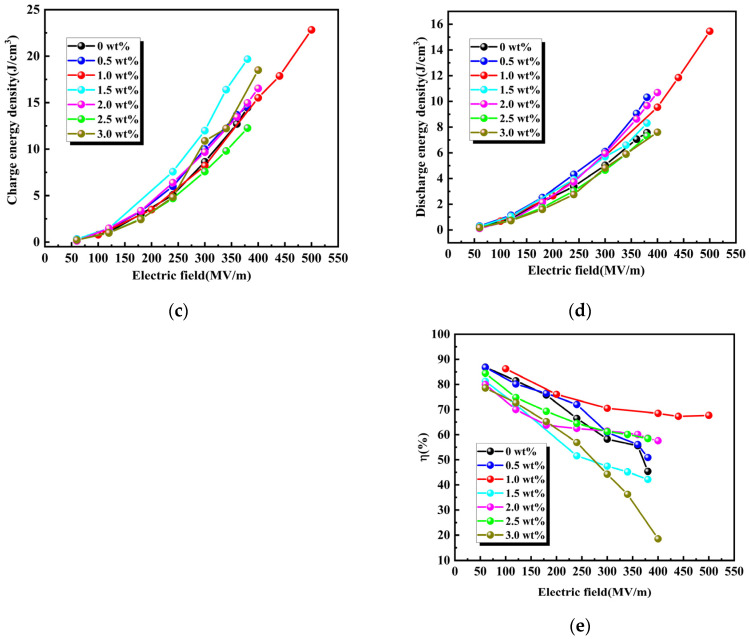
Energy storage performance of BT/PVDF composite with varying BT NPs: (**a**) P_r_; (**b**) P_max_; (**c**) charge; (**d**) discharge energy storage density; (**e**) efficiency.

Figure 9a–e present a comprehensive comparison of the ferroelectric and energy storage properties, including remnant polarization (P_r_), maximum polarization (P_max_), charge (U_charge_), discharge (U_dis_) energy storage density, and efficiency (η). As shown in Figure 9a,b, both P_r_ and P_max_ increase with the applied electric field. Among the tested compositions, the 1.0 wt% BT composite achieves an optimal balance, maintaining the lowest Pr (1.5 μC/cm^2^ at 500 MV/m) while achieving P_max_ of 8.7 μC/cm^2^. In contrast, the 3.0 wt% BT composite achieves P_r_ of 2.9 μC/cm^2^ under a lower breakdown field of 400 MV/m. The U_charge_ and U_dis_ of BT/PVDF are depicted in Figure 9c,d. It can be shown that the U_dis_ of BT/PVDF with 1 wt% BT reaches 15.5 J/cm^3^ at 500 MV/m, which is about two times higher than that of pure PVDF (7.6 J/cm^3^ at 380 MV/m). Consequently, BT NPs have a pronounced effect on energy storage performance, with even minimal loading significantly increasing the breakdown strength and polarization of BT/PVDF composite thick films. The ferroelectric and energy-storage properties are mainly attributed to the uniform dispersion of low-content BT NPs, which act as efficient heterogeneous nucleation sites that enhance PVDF crystallization and reduce leakage conductance from filler agglomeration. The 1.0 wt% BT NPs composite has the highest Pmax and the lowest Pr among the others. According to formulas (1) and (2), higher energy storage efficiency indicates smaller energy dissipation, higher utilization rates, and decreased losses. Achieving optimal energy storage efficiency requires materials with low residual polarization strength and strong dielectric-loss suppression, thereby reducing energy dissipation and enhancing output efficiency. This means that the material’s high energy efficiency makes it highly reversible at this composition, with irreversible energy loss significantly suppressed. As displayed in Figure 8e, the 1.0 wt% composite retains a high efficiency of 68% even at 500 MV/m, while the 3.0 wt% counterpart drops drastically to 19% at 400 MV/m, which is associated with the aggravated leakage current and larger remanent polarization at higher filler content. The superior energy efficiency of the 1 wt% BT/PVDF composite reduces thermal dissipation during charge–discharge cycles, thereby improving reliability and stability under high electric fields.

(2) $$\eta =\frac{U_{discharge}}{U_{charge}}$$

## 4. Conclusions

This study systematically investigates polyvinylidene fluoride (PVDF)-based composites incorporated with barium titanate (BaTiO_3_, BT) at different loadings. The chemical composition and phase structure of the BT/PVDF composites were comprehensively represented via X-ray diffraction (XRD), Fourier transform infrared (FT-IR) spectroscopy, and Raman spectroscopy. As the BT NPs content increased, the crystallinity of the BT/PVDF composites gradually decreased, which effectively inhibited the formation of both α-phase and β-phase PVDF. Further, the influence of BT NPs loading on the crystal structure and breakdown strength (U_dis_) of the composite thick films was systematically examined. Experimental results demonstrate that the 1.0 wt% BT/PVDF composite, at the optimal BT NPs loading, achieves optimal energy storage performance: a maximum energy storage density of 15.5 J/cm^3^ under an electric field of 500 MV/m, accompanied by an energy storage efficiency of 68%. With its excellent flexibility and superior energy storage capabilities, the BT/PVDF composite thick film exhibits considerable potential for practical applications in advanced human–machine interaction flexible electronic devices.

## Figures and Tables

**Table 1:** FT-IR characteristic peaks correspond to the two different crystalline forms of PVDF.

Phase	Wavenumber (cm^−1^)							
α phase	490	532	573	614	763	796	851	916
β phase	510	844	--	--	--	--	--	--

**Table 2:** Comparison of energy storage performance of PVDF-based composites.

Composites	E_b_ (MV/m)	U_d_ (J/cm^3^)	η (%)	Reference
3.0 vol% BT-PVDF	420	11.5	64	[35]
30.0 vol% BT-PVDF	200	6.7	50	[36]
2.0 wt% BT-PVDF	234	2.9	75	[37]
10.0 vol% BT-PVDF	275	6.1	72	[33]
1.0 wt% BT-PVDF	500	15.5	68	This work

## Data Availability

The original contributions presented in this study are included in the article. Further inquiries can be directed to the corresponding author.
